# Dampening of Hyperexcitability in CA1 Pyramidal Neurons by Polyunsaturated Fatty Acids Acting on Voltage-Gated Ion Channels

**DOI:** 10.1371/journal.pone.0044388

**Published:** 2012-09-25

**Authors:** Jenny Tigerholm, Sara I. Börjesson, Linnea Lundberg, Fredrik Elinder, Erik Fransén

**Affiliations:** 1 Department of Computational Biology, School of Computer Science and Communication, KTH Royal Institute of Technology, Stockholm, Sweden; 2 Stockholm Brain Institute, KTH Royal Institute of Technology, Stockholm, Sweden; 3 Division of Cell Biology, Department of Clinical and Experimental Medicine, Linköping University, Linköping, Sweden; Georgia State University, United States of America

## Abstract

A ketogenic diet is an alternative treatment of epilepsy in infants. The diet, rich in fat and low in carbohydrates, elevates the level of polyunsaturated fatty acids (PUFAs) in plasma. These substances have therefore been suggested to contribute to the anticonvulsive effect of the diet. PUFAs modulate the properties of a range of ion channels, including K and Na channels, and it has been hypothesized that these changes may be part of a mechanistic explanation of the ketogenic diet. Using computational modelling, we here study how experimentally observed PUFA-induced changes of ion channel activity affect neuronal excitability in CA1, in particular responses to synaptic input of high synchronicity. The PUFA effects were studied in two pathological models of cellular hyperexcitability associated with epileptogenesis. We found that experimentally derived PUFA modulation of the A-type K (K_A_) channel, but not the delayed-rectifier K channel, restored healthy excitability by selectively reducing the response to inputs of high synchronicity. We also found that PUFA modulation of the transient Na channel was effective in this respect if the channel's steady-state inactivation was selectively affected. Furthermore, PUFA-induced hyperpolarization of the resting membrane potential was an effective approach to prevent hyperexcitability. When the combined effect of PUFA on the K_A_ channel, the Na channel, and the resting membrane potential, was simulated, a lower concentration of PUFA was needed to restore healthy excitability. We therefore propose that one explanation of the beneficial effect of PUFAs lies in its simultaneous action on a range of ion-channel targets. Furthermore, this work suggests that a pharmacological cocktail acting on the voltage dependence of the Na-channel inactivation, the voltage dependences of K_A_ channels, and the resting potential can be an effective treatment of epilepsy.

## Introduction

Epilepsy is a severe neurological disorder which is characterized by spontaneous recurrent seizures. Many factors have been linked to the etiology, among them ion channels. Voltage-gated ion channels are crucial for generating and regulating neuronal excitability. Their pivotal importance is evidenced by multiple channel mutations inducing hyperexcitability and epilepsy in humans [1], [2]. As a rule of thumb, opening of voltage-gated sodium (Na) channels increases excitability while opening of voltage-gated potassium (K) channels reduces excitability. Thus, several gain-of-function mutations in Na channels [1] as well as loss-of-function mutations in both delayed rectifier (K_DR_) and A-type K (K_A_) channels [3]–[7] are associated with epilepsy.

The strong connection between voltage-gated ion channels and neuronal activity makes ion channels an attractive pharmacological target for anticonvulsive substances. The traditional pharmacological strategy is to reduce excitatory Na currents by targeting the ion conducting pore [8]–[10]. However, despite the great number of antiepileptics on the market, about 20–30% of patients with epilepsy respond incompletely to drug treatment [11]. Lack of therapeutic effects in many patients in combination with adverse effects [12], [13] motivates the search for new antiepileptic drugs, new targets, and new pharmacological mechanisms.

An epileptic seizure has a rich repertoire of events. One pronounced feature during epileptogensis is highly synchronized neuronal activity [14]–[17]. Synchronous input is very powerful in activating neurons [18]–[20], and therefore an enhanced neuronal response can be part of the pathology. In a previous study [21], we showed that highly synchronized activity is suppressed by the K_A_ channel in dendrites, which therefore may function as a protective mechanism against hyperexcitability. In epilepsy, the K_A_ current may not be strong enough to compensate for the excitability changes due to the pathology. Thus, substances changing the activity of channels involved in suppressing cellular responses to synchronicity may be a powerful way to prevent epileptic seizures.

**Figure 1:**
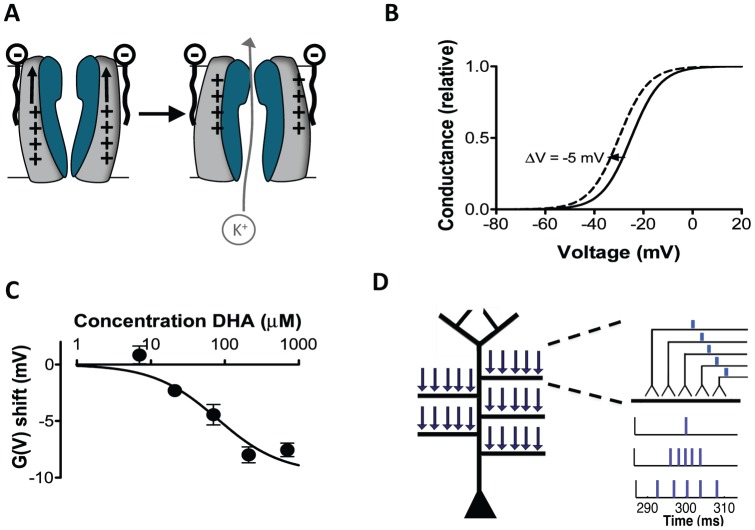
Conceptual background to the computational approach. (A) Schematic illustration of PUFA modulation of ion channel voltage dependence. (B) The continuous line schematically illustrates the conductance *versus* voltage curve for a voltage-gated ion channel. The dashed line illustrates a PUFA-induced negative shift of the voltage dependence. (C) Dose-response curve for the PUFA (docosahexaenoic acid  =  DHA) induced shift of the voltage dependence of the Shaker channel expressed in Xenopus oocytes. pH  = 7.4. Figure modified after Xu et al. (2008) (D) Schematic figure of the CA1 pyramidal cell and the placement of the synaptic inputs used in the simulations. The five medial to distal oblique dendrites were located 424, 500, 544, 635, and 715 µm from the soma. On each oblique dendrite, five synaptic inputs were placed (arrows). The first input cycle was set to have a midpoint at 300 ms, providing an initial period of baseline membrane potential. To represent inputs of different synchronicity levels, input arrival times were jittered using normal distributions of different widths (standard deviations).

Polyunsaturated fatty acids (PUFAs) are suggested as important antiepileptic substances in the fat-rich ketogenic diet used as an alternative epilepsy treatment in children [22]–[25]. The mechanism of the ketogenic diet is largely unknown, but PUFAs directly target a wide range of ion channels, including Na and K channels e.g. [24], [26]. The proposed mechanism for the modification suggests that the partly negatively charged lipophilic molecules target the lipid bilayer close to the positively charged voltage sensor of ion channels and electrostatically activate the channel and open the ion-conducting pore (Fig. 1A) [27]–[29]. This interaction with the voltage sensor leads to a modified voltage dependence, so that the channel opens at more negative voltages (Fig. 1B). Figure 1C shows the concentration dependence of the PUFA-induced shift of K channel activation (see figure legend for further details). In Na channels PUFAs instead shift the voltage dependence of channel inactivation.

The objective of this study was to investigate PUFAs as reducers of excessive neuronal responses to synchronous input, and thereby as possible anticonvulsive substances. To experimentally test the importance and contribution of each PUFA-induced channel modulation separately is however very difficult as specific antagonists of the modulation are unavailable. The evaluation is further complicated by some effects being predicted to oppose effects of other [24], [26]. In this study we therefore turned to a computational model of a pyramidal neuron in hippocampal area CA1 (see Fig. 1D), a brain region commonly involved in epileptic seizures [17], [30]. During a simulation we activated five synaptic inputs contacting five oblique dendrites of the CA1 neuron. In different runs, the synchronicity between the inputs varied from completely simultaneous (i.e. synchronized) to relatively desynchronized. The number of produced action potentials was used as a measure of excitability.

To study hyperexcitability we implemented two cases of ion-channel alterations associated with epileptogenesis. The first case comprised enhanced Na currents [31] and the second case a rundown in the K_A_ conductance [3], [32]. In the two pathological models we subsequently investigated how PUFAs modulate the cellular response by altering the activity of voltage-gated ion channels. We focused on three prominent channels: a Na channel, an A-type K (K_A_) channel, and a delayed-rectifier K (K_DR_) channel. PUFA modulation of the Na channel was implemented as a hyperpolarizing shift of the steady-state inactivation curve as reported in human and rat cardiac Na channels and rat CA1 Na channels [33]–[35]. For further information on ion channel modulation and its implementation, see the Models section. This leads to a reduced Na current [35], [36]. We implemented PUFA modulation of K_A_ as a hyperpolarizing shift of the steady-state activation curve [24], leading to an increased K current, and assumed K_DR_ to be similarly affected by PUFAs. Furthermore, the resting membrane potential is altered by PUFAs [37]–[39] and possibly also by other ketogenic diet agents [40], [41] that may act on ion channels such as the ATP-dependent K channel, the h-channel, or the K-conducting M-channel. We therefore also tested the impact of altered resting potential on excitability. In the second part of this study we investigated the effect on the pathological cell models when simultaneously shifting the steady-state activation and inactivation curves equally much or when simultaneously affecting several ion channels.

The goal was to restore a physiological, non-epileptogenic, neuronal response. The studies showed that modifications of the Na channel, the K_A_ channel, or the resting potential were effective ways to normalize a pathological behaviour. Thus, PUFAs and substances acting through similar mechanisms could be potent anticonvulsive substances by modulating the activity of multiple ion channels and thereby reducing the neural response to synchronous input.

## Results

### The cellular response to highly synchronized input is suppressed

To study the effects of PUFAs on neuronal excitability, we performed computer simulations using the CA1 pyramidal neuron model by Migliore et al. [42]. The cell model, based on a morphologically reconstructed neuron, includes a transient Na current, a persistent K_DR_ current, a transient K_A_ current, and a pacemaker h current. CA1 is one of the key regions implicated in temporal lobe epilepsy and the pyramidal neuron is proposed to be the primary neuron type generating fast ripples [15], a highly synchronized ensemble activity associated with seizures. As epilepsy ultimately is a network phenomenon, and because communication between neurons is performed via spikes, measuring spike production is one way of addressing excitability on a single neuron level. In this work, we therefore measured the number of spikes produced and used this as a measure of responsiveness to the input provided.

**Figure 2:**
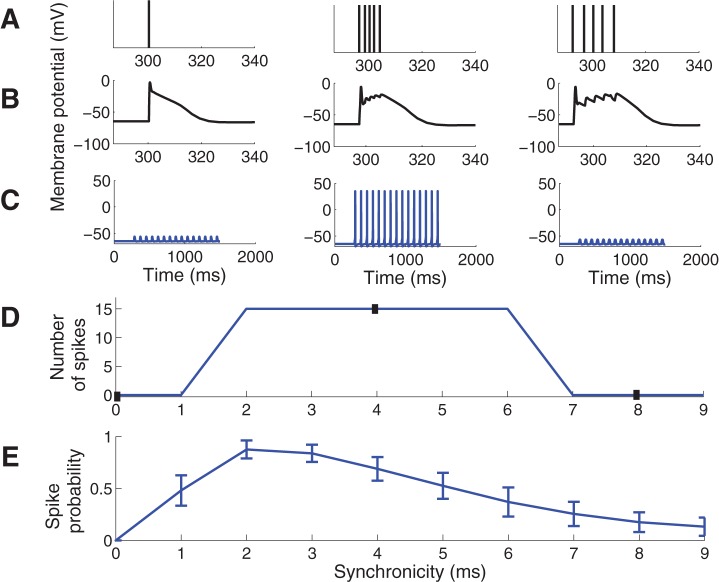
Spike-response diagram for the control model for inputs of different synchronicity. (A–C) The left column shows simulation results with synchronicity of 0 ms, middle column of 4 ms, and right column of 8 ms. The first input cycle was set to have a midpoint at 300 ms, providing an initial period of baseline membrane potential. (A) Temporal distribution of synaptic inputs. Each bar corresponds to one synaptic input. The 5 inputs are superimposed in the left panel. (B) The membrane potential (EPSP) at one of the input sites. (C) The membrane potential in the soma following 15 input repetitions at 12 Hz. (D) Number of spikes generated for different synchronicity levels of the input. Closed boxes correspond to synchronicity levels and spike counts shown in C. Note the absence of spikes for high synchronicity levels (0 and 1 ms). (E) Spike probability when the input was chosen from a randomly sampled normal distribution. Error bars indicate the standard deviation from 50 simulations.

The timing of arrival of each input was adjusted to obtain stimuli of different synchronicity (Fig. 1D, Fig. 2A) where a larger jitter of the inputs (Fig. 2A right) represents less synchronized input, and zero time difference (Fig. 2A left) corresponds to perfectly synchronized input. Passive biophysics would predict that highly synchronized input should be the most effective input to activate a neuron. However, this is not the case here since highly synchronized input (<2 ms) is suppressed (Fig. 2B–D). This is consistent with experimental data where inputs were given within a short time window, as further discussed in the Discussion, and with our previous simulation results using a single-input site on a medial location of the apical dendrite [21]. Figure 2E shows the spike probability when the input jittering was randomly sampled from a normal distribution rather than sampled uniformly as in Figure 2D. The main difference between the two sampling methods was that the random sampling produced a smoother curve while qualitative characteristics were similar, in particular for the highly synchronized inputs which are our main interest.

### Pathological models produce increased cellular response for highly synchronized input

A range of ion channels have been linked to epileptogenesis, particularly Na and K channels. Several Na channel gene mutations causing an increased open probability have been linked to epilepsy [1], and a study on kindling epileptogenesis observed a 22% increase in the peak Na current [31]. Thus, to implement a Na channel pathology, we increased the Na conductance by 22%. This model is referred to as the “increased Na current pathology”. The involvement of the K_A_ channel in epileptogenesis has been studied in both animal models and in human tissue [3], [32], [43], [44]. Some of these studies show a downregulation of the gene expression or the current of K_A_ [44]. To implement a K_A_-channel pathology, we decreased the K_A_ conductance by 50% [3], [32]. This model is referred to as the “decreased K_A_ current pathology”. As we are unaware of any cases of combined Na and K channel mutations, we have not studied this combined case.

**Figure 3:**
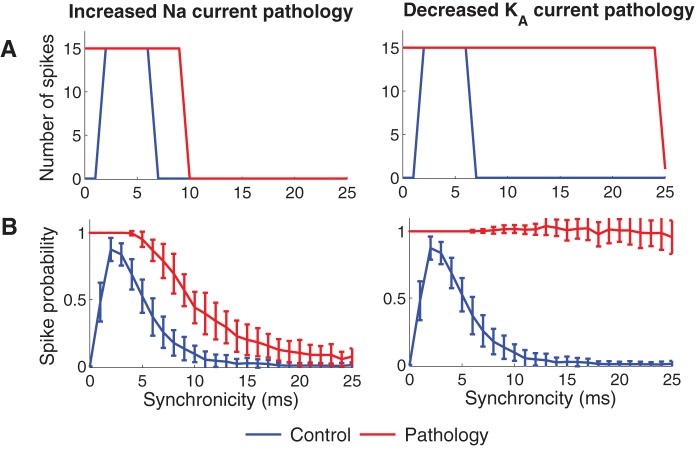
Pathological ion channel alterations generate hyperexcitable cell models. The figure shows the number of spikes generated when different levels of synchronized input were provided to the cell. The left column shows the increased Na current pathology (22% increase in Na conductance). The right column shows the decreased K_A_ current pathology (50% decrease in K_A_ conductance). (A) Deterministic sampling model for the inputs and (B) random sampling model for inputs. Error bars indicate the standard deviation from 50 simulations. The spike counts for the control model (blue) and pathological models (red) are shown. Note that the pathological models do not reduce the cellular response to highly synchronized inputs (0–1 ms). Furthermore the decreased K_A_ current pathology shows hyperexcitability also for lower synchronicity levels (7–24 ms).

Figure 3A shows the output spike responses of the control model (blue lines) and the two pathological models (red lines) for different degrees of input synchronicity. Both pathological models clearly displayed hyperexcitability which is consistent with our previous study [45]. For inputs of low synchronicity (>6 ms; large jitter values), the decreased K_A_ current model showed particularly large increases in output excitability. The low-synchronicity input may be regarded as background input and provides a measure of general excitability. More importantly, highly synchronized inputs (<2 ms; small jitter values), with particular interest for epileptogenesis (see Discussion), led to increased responses in both pathological models. Figure 3B shows the spike probability using random sampling of the input jittering. The result is similar as for the deterministic input model in Figure 3A, in particular at the highest levels of synchronicity which constitute the region of interest in the present study. As random sampling requires a great number of simulations to be representative, we will in the following use the deterministic uniformly sampled distribution.

### Pharmacological correction of pathological models

**Figure 4:**
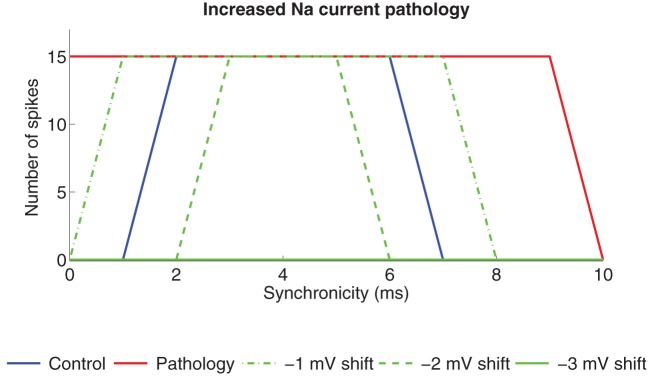
Modulation of K_A_ channels reduces the spike activity in the increased Na current pathology model. Spike activity for different synchronicity levels is reduced when the steady-state activation curve of the K_A_ channel is shifted in the negative direction along the voltage axis. When the shift was −3 mV (or higher) the cell did not produce any spike at any level of synchronized input and was therefore regarded as pathological.

The pathological models of the pyramidal neuron in CA1 generate excess spiking when input of different synchronicity is provided (Fig. 3). The main objective of this study was to correct the pathologies by reducing the excess spiking for high-synchronicity levels (<2 ms). This region of synchronicity level, and the level at which spiking switches from a few spikes to strong spiking is thereby of key interest and is therefore shown for each case below as a spike graph. To correct the pathologies we simulated the experimental effects of PUFAs on the Na, K_DR_, and K_A_ channels. We also studied the effect of an altered resting membrane potential. For more information of the modulation of the ion channels and the resting membrane potential see the Models section. The correction strategy is exemplified in Figure 4, illustrating three attempts to correct the increased Na current pathology by shifting the steady-state activation curve of the K_A_ channel by −1, −2, or −3 mV along the voltage axis. The increased K_A_ channel activity, caused by the negative shift, reduced the number of spikes. For shifts of −1 and −2 mV, the reduction in the number of spikes is selective for the highest and lowest levels of synchronicity. A shift of −3 mV abolished output activity for all synchronicity levels. Thus, a shift between −1 mV and −2 mV generates to best correction to the original, healthy neuron. In the following we will define the pathological model as functionally corrected if zero spikes are generated for completely synchronized input (0 ms) and the maximum number of spikes (15) at synchronicity level 2 ms. Thus, for each correction there exists a therapeutic interval where these criteria are satisfied.

#### Correcting the increased Na current pathology

**Figure 5:**
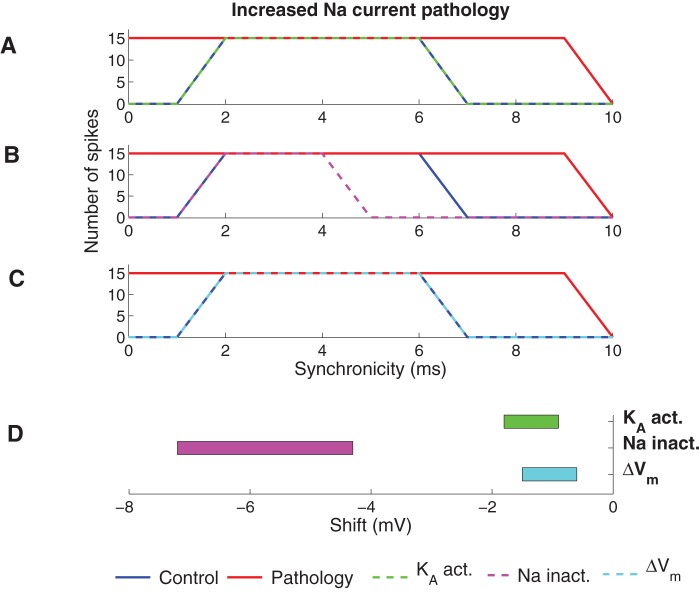
Effects of PUFA on the increased Na current pathology model. (A–C) The spike activity for the functionally corrected model when (A) K_A_ is modulated by shifting the steady-state activation curve (−1.6 mV), (B) Na is modulated by shifting the steady-state inactivation curve (−6.7 mV) and (C) the resting membrane potential was shifted (−1.1 mV). (D) The interval of the shift of the steady-state curves or resting membrane potential where the model was functionally corrected (generated zero spikes for synchronicity level 0 ms and 15 spikes for synchronicity level 2 ms).

Figure 5 shows the increased Na current pathology model corrected by the experimentally described PUFA modulation of the K_A_ channel, the Na channel, and the resting membrane potential, respectively. Modulation of the K_A_ channel by shifting the steady-state activation curve by −1.6 mV (Fig. 5A) or the resting potential by −1.1 mV (Fig. 5C) restored the spike activity for both high (<2 ms) and low (>6 ms) synchronicity levels, while modulation of the Na channel by shifting the steady-state inactivation curve by −6.7 mV (Fig. 5B) suppressed the spike activity for high and low synchronicity levels but also suppressed the activity for some intermediate (5–6 ms) synchronicity levels. PUFA-induced shifts of the K_DR_ channel steady-state activation curve (data not shown) were unable to correct the pathological model. When the shift was large enough (−27.2 mV) to abolish spikes for high synchronicity levels (0 ms and 1 ms) it was not able to produce 15 spikes at 2-ms synchronicity. A functionally corrected model should be robust in the sense that small alterations of parameter values (here PUFA-induced shifts of the channels' voltage dependences or the resting potential) should be tolerated without reintroducing pathological spiking. But, at the same time, the model should not be totally insensitive to changes of a parameter presumed to play a role in correcting the pathology. The models were therefore analyzed further by measuring the parameter interval within which the model was classified as functionally corrected, that is no spikes at 0-ms synchronicity and 15 spikes at 2-ms synchronicity (Fig. 5D). Smaller shifts were needed for the K_A_ channel (−0.9 to −1.8 mV) than for the Na channel (−4.3 to −7.2 mV).

#### Correcting the decreased K_A_ current pathology

**Figure 6:**
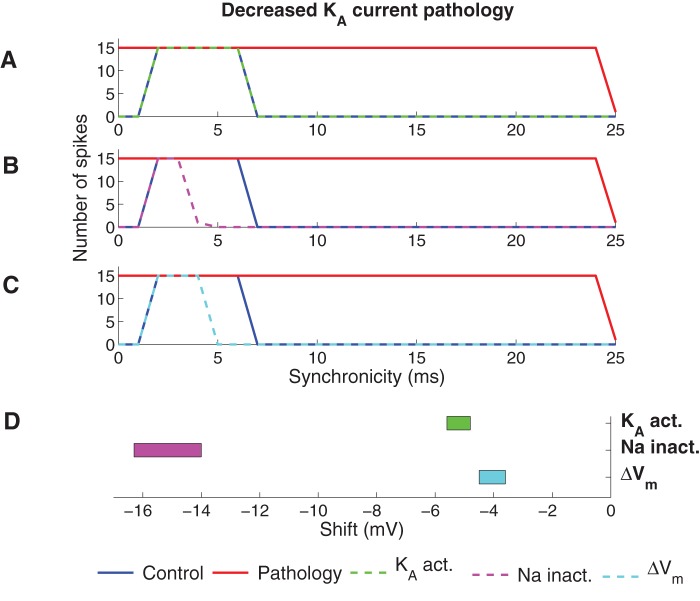
Effect of PUFA on the decreased K_A_ current pathology model. (A–C) The spike activity for the functionally corrected model when (A) K_A_ is modulated by shifting the steady-state activation curve (−5.4 mV), (B) Na is modulated by shifting the steady-state inactivation curve (−15.4 mV) and (C) the resting membrane potential was shifted (−4.2 mV). (D) The intervals of the shift of the steady-state curves or resting membrane potential where the model was functionally corrected (generated zero spikes for synchronicity level 0 ms and 15 spikes for synchronicity level 2 ms).

Figure 6A–C shows the best correction of the decreased K_A_ current pathology for PUFA modulation of the K_A_ channel, the Na channel, and the resting membrane potential, respectively. PUFA modulation of the K_A_ channel perfectly restored normal output activity, while modulation of the Na channel and the resting potential were not able to compensate the output activity at both low- and high-synchronicity at the same time; when functional corrections were obtained at synchronicity levels of 0–2 ms, the correction was too large at intermediate synchronicity of 4–6 ms. As for the increased Na current pathology model, shifting the voltage dependence of the K_DR_ channel did not correct the K_A_ current pathology (data not shown). Figure 6D shows the interval of the modulatory shift within which output was classified as functionally corrected. Also for the decreased K_A_ current pathology model, the smallest shifts required to correct the model were found for K_A_ and the resting membrane potential.

### Impact of shifting both steady-state activation and inactivation curves simultaneously

**Figure 7:**
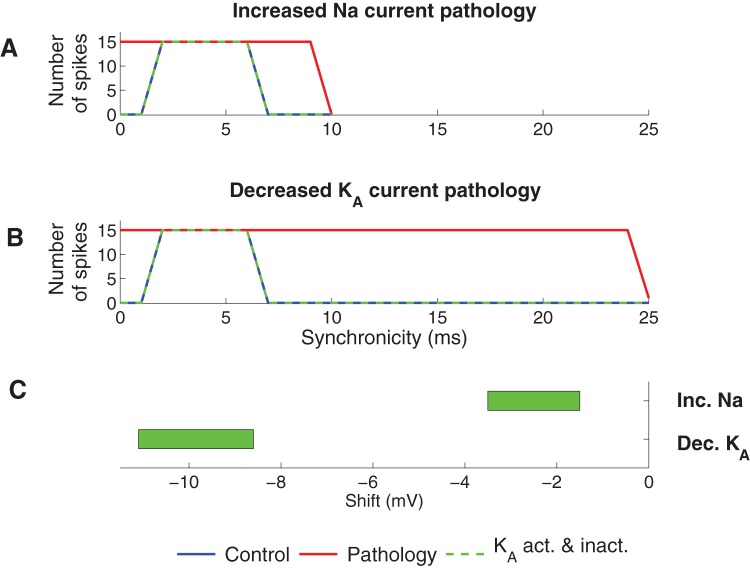
Effect of PUFA when the steady-state activation and inactivation of K_A_ is equally modulated. (A) Spike activity for the functionally corrected model of the increased Na current pathology model. (B) Spike activity for the functionally corrected model of the decreased K_A_ current pathology model. To functionally correct the two pathologies the steady-state activation and inactivation curves of K_A_ were shifted −3.2 mV (increased Na current pathology) and −11 mV (decreased K_A_ current pathology). (C) Shift regions for the K_A_ steady-state curves where the pathologies were functionally corrected (generated zero spikes for synchronicity level 0 ms and 15 spikes for synchronicity level 2 ms).

It is known that PUFAs affect both steady-state activation and inactivation of some Na channels [46]. Furthermore, inactivation of K_A_ is tightly coupled to activation [47], meaning that a shift in the channel's steady-state activation curve is expected to result in an equally large shift of the steady-state inactivation curve. In this section we consequently extended the study by allowing PUFAs to shift both steady-state activation and inactivation curves by equal amounts for Na and K_A_ channels. These simulations were not applicable to the K_DR_ channel since it is not capable of fast inactivation. If both the steady-state activation and inactivation curves of the Na channel were shifted, the Na current increased and as a consequence neither of the pathological models could be functionally corrected (data not shown). Conversely, both pathological models could be functionally corrected when the two steady-state curves of the K_A_ channel were equally shifted (Fig. 7).

### The combined effect of PUFA on several ion channels

**Figure 8:**
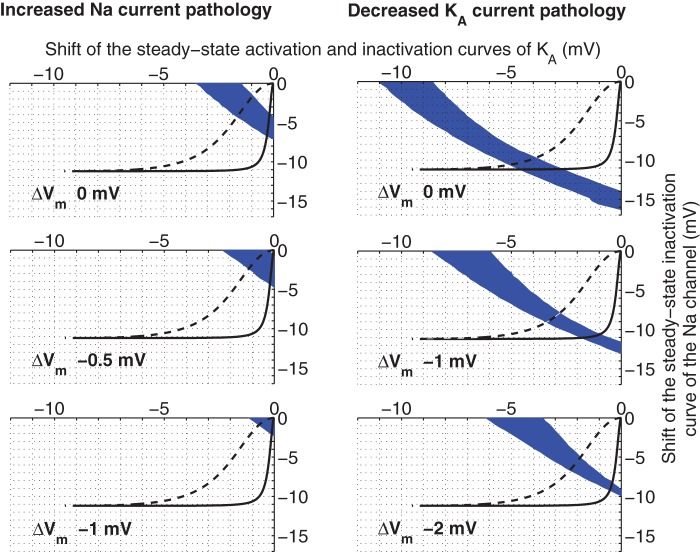
Regions of functionally corrected models when PUFA is affecting the Na and K_A_ channels, and the resting membrane potential simultaneously. These nomograms indicate the areas (blue) where the pathological models are functionally corrected for different degrees of resting potential adjustments (0, −0.5, −1.0 mV top to bottom for the increased Na current pathology, and 0, −1.0, −2.0 mV top to bottom for the decreased K_A_ current pathology). PUFA was modelled as a shift of the steady-state inactivation curve of the Na channel, shifts of steady-state activation and inactivation curves of the K_A_ channel, and a hyperpolarizing shift of the membrane potential. The black lines are solutions for Eq. 2 in the main text for the cases of the K_A_ channel and the Na channel. Each point in the line represents the shifts for Na inactivation and K_A_ activation respectively for a particular concentration. The dashed line is the corresponding line for CA1-corrected K_A_ data.

Above, we have studied the effects of modulating one ion channel at a time in two different pathological models. In the next step we explored the three-dimensional space of the combined effect of PUFA modulation of K_A_ and Na channels, and the resting membrane potential. Figure 8 shows three slices of the explored three-dimensional space for both the increased Na current pathology and the decreased K_A_ current pathology models. PUFA was modelled as 1) a shift of the steady-state inactivation curve of the sodium channel, 2) combined shifts of steady-state activation and inactivation curves of the K_A_ channel, and 3) an alteration of the membrane potential. The solutions of functionally corrected models are found in the blue areas. Interestingly, but not surprisingly, smaller effects on the targets are needed for functional correction if all three effects are included simultaneously.

### Therapeutic intervals

Based on experimentally obtained dose-response curves described in the Models section (Eq. 2) it is possible to calculate the therapeutic concentration intervals for effects on the steady-state Na inactivation and K_A_ activation curves. The dose-response curve given for Na inactivation is from CA1 neurons (explored in the present investigation). In contrast, the dose-response curve for the K_A_ activation is from the Shaker K channel expressed in *Xenopus* oocytes, which is known to underestimate the efficacy of pharmacological substances [48]. Therefore, in the quantitative evaluation below we will give alternative therapeutic intervals assuming a 10 times higher affinity in CA1 neurons than in the *Xenopus* oocytes (referred to as CA1 affinity). The therapeutic interval for PUFA affecting the resting potential is not possible to calculate because of limited experimental data available. Instead, we will estimate the upper limit of the therapeutic interval (see Models section for further details). Table 1 summarizes the therapeutic interval needed to restore the pathological models.

**Table 1:** The therapeutic intervals of the concentration needed to functionally correct two pathological models.

	Increased Na current pathology		Decreased K_A_ current pathology	
	Shift (mV)	Concentrations (μM)	Shift (mV)	Concentrations (μM)
**Na_inact._**	4.3–7.2	1.7–2.8	14.0–16.3	-
**K_A,act._**	0.9–1.8	8.2–18 (0.82–1.8*)	4.8–5.6	79–110 (7.9–11*)
**K_DR,act._**	-	-	-	-
**ΔV_m_**	0.6–1.5	<3.9	3.6–4.5	∼10
**Na_act._ + Na_inact._**	-	-	-	-
**K_A,act._ + K_A,inact._**	1.5–3.5	15–45 (1.5–4.5*)	8.6–11.1	>680 (>68*)
**Na_inact._ + K_A,act._ +K_A,inact._ +ΔV_m_**	Varies	<1.5 (<1.1*)	Varies	<4.8 (<3.5*)

* denotes CA1 affinity.

For the increased Na current pathology model, the therapeutic interval for PUFA when only K_A_ activation is affected is 8.2–18 μM (0.82–1.8 μM assuming CA1 affinity, see above). If only Na inactivation is affected, the interval is 1.7–2.8 μM. The therapeutic interval for PUFA acting on the resting potential is <3.9 μM. Moreover, if PUFA acts on both steady-state inactivation and activation curves for the K_A_ channel the therapeutic interval is 15–45 μM (1.5–4.5 μM assuming CA1 affinity). Interestingly, this means that the therapeutic interval widens from 8.2–18 μM (factor of 2.2) to 15–45 μM (factor of 3), resulting in a more stable correction even though higher concentrations are required. Furthermore, if PUFA acts simultaneously on the steady-state Na inactivation, steady-state K_A_ activation and inactivation, and the resting potential, then the therapeutic interval is <1.5 μM (<1.1 μM assuming CA1 affinity).

For the decreased K_A_ current pathology model, the therapeutic interval for PUFA when only K_A_ activation is affected is 79–110 μM (7.9–11 μM assuming CA1 affinity). If both the steady-state inactivation and the activation curves are modulated it is above 680 μM (>68 μM assuming CA1 affinity). When PUFA only acts on Na inactivation, modulatory effects are not sufficient and no correction is obtained. Furthermore, the therapeutic interval for PUFA acting on the resting potential is ∼10 μM. Moreover, if PUFA acts simultaneously on the steady-state Na inactivation, steady-state K_A_ activation and inactivation, and the resting potential, then the therapeutic interval is <4.8 μM (<3.5 μM assuming CA1 affinity). Thus, strikingly low PUFA concentrations are in several cases adequate to rescue the pathologies. The amount of PUFA required is well within the estimated PUFA concentration range in cerebrospinal fluid during ketogenic diet treatment [22], [24]. This highlights the pharmacological potency of shifts in channel voltage-dependencies or the cellular resting potential.

## Discussion

In this work we have modelled effects of PUFAs on excitability in a CA1 pyramidal cell. The rationale behind this was to explore a possible mechanism for the ketogenic diet used in the treatment of epilepsy. One important factor contributing to its anticonvulsive effect is believed to be direct ion-channel effects due to elevations in PUFA levels. We have investigated whether experimentally reported PUFA-induced changes in ion channel characteristics can reverse epileptogenic hyperexcitability and thereby normalize neuronal excitability. Altogether our results show that small alterations either from shifting steady-state curves or resting membrane potential is effective in reducing hyperexcitability, particularly for high synchronicity input. Table 1 summarizes the findings of the present investigation.

### Selective modulation of the response to synchronous input may minimize adverse effects

Our study has focused on suppression of highly synchronized input as an effective means to reduce excitability related to epileptogenesis. Epileptogenic activity, and in particular fast ripples, is characterized by spiking at high synchronicity levels [14]–[17]. Such activity will at target cells produce synchronous compound EPSPs characterized by fast rise times, large amplitudes and short duration. *In vivo*, action potentials most commonly arise from brief dendritic depolarisations of this kind [19], [49]. Moreover, synchronous input *in vitro* is the most effective input to produce spikes [18]–[20]. We therefore argue that selective reduction of the response to synchronous input might be more beneficial than reduction of spiking activity in general which might be causing the sedative adverse reactions commonly reported for anticonvulsive agents. To us it was an unexpected finding that a shift of the resting potential reduced the pathological excitability without leading to a decrease of the general excitability. Our interpretation is that the selective effect is mediated indirectly by the voltage dependence of K_A_, in particular its steady-state inactivation.

### Synchronicity and network activity

We have in this work studied how neuronal responses to synchroneous input can be modulated by regulation of the properties of K_A_. Our results are consistent with experimental studies using inputs given within a short time window [58]–[60]. We further argue that when this suppression of spike generation occurs on all neurons, it provides a dampening mechanism on network activity. Factors that regulate the level of synchronicity in the network have been extensively studied. On the single neuron level, phase response studies have provided information on how dendritic ionic conductances may affect whether the neuron fires in phase with the input or out of phase [61], [62] and thus whether synchronicity is enhanced or suppressed. Moreover, network synchronicity was initially studied in pairs of neurons [63] and in highly reduced systems [64]. On a network level, it was shown that a classification of neuron types as integrators or resonators provides fundamental information on how neurons will respond to excitatory or inhibitory input and how this will shape activity in the network into synchroneous or asynchroneous/independent collective states. More recently, the role of intrinsic ionic currents in network synchronicity has been highlighted [65], [66].

### The best candidate targets

To rescue the increased Na current pathology, there are a number of reasonable target candidates. Acting on Na inactivation, K_A_ activation and inactivation, or resting membrane potential requires roughly 1–5 μM of PUFA (assuming CA1 affinity). Combined effects on all these targets lower the concentration even further. To rescue the decreased K_A_ current pathology we are limited to effects on the resting potential or combinations including effects on the resting membrane potential, given that we are not able to act separately on the K_A_ activation and if we believe that the increase in PUFA concentrations during the ketogenic diet does not exceed 30 µM, see [24]. Combined effects on all three targets lower the PUFA concentration required to <4 μM. In contrast to these successful modulations, PUFA modulation of the K_DR_ channel was unable to correct any of the two pathologies. Our interpretation is that for an ion channel to contribute significantly to the occurrence of a spike at high synchronicity levels, it has to be fast enough to affect the EPSP before it reaches the spike threshold. K_DR_ may nevertheless have effects on the spike threshold and slower depolarisations.

### Combined effects on several ion channels as future pharmacological approach?

Combined effects on several ion channels means that beneficial effects are achieved with lower concentrations of the modulator. These simulations highlight the possible usefulness of a cocktail of pharmacological compounds in epilepsy treatment, each with high specificity and affecting different ion channel targets. For instance could M-channel openers like ZnPy [50], retigabine [51], acrylamide (S)-2 [52] and NH29 [53], which are expected to hyperpolarize the resting potential, potentially be combined with compounds targeting K_A_ channel voltage dependence or Na channel inactivation for a more effective antiepileptic effect. Our simulations further imply that K_A_ modulating drugs would act beneficial even when steady-state inactivation is affected similarly as steady-state activation, which would be the most biological relevant modulation of K_A_ [47]. In contrast, for Na channels a more selective modulation of only the inactivation is preferable. Indeed, the modulation pattern generally seen for PUFAs on Na channels is dominated by the effect on Na channels inactivation.

## Models

In this study we investigated the effect of PUFAs on hyperexcitable pathological neurons using computational methods. The reason for using a computational strategy is that the effects on different ion channels can be isolated and evaluated on its own, and that the effect of synaptic input with different levels of synchronicity can be studied in detail.

### The cell model

All simulations were performed using the simulator NEURON [54]. The neuron model was based on the work by Migliore et al. [42], ModelDB accession number 87535. It is a detailed compartmental model of a CA1 pyramidal cell with 474 compartments. The cell model includes a transient Na current, a persistent K_DR_ current, a transient K_A_ current, and a pacemaker h current. The ion channels were described by Hodgkin-Huxley dynamics. The resting potential of the neuron was set to -65 mV. To this published model we added synaptic input of different synchronicity levels. We used five synaptic inputs at five dendritic branches located at distances of 424, 500, 544, 635, and 715 μm from the soma (Figure 1D). This gives a total of 25 synaptic inputs. Each synapse was stimulated with a frequency of 12 Hz. The simulation was run for 1500 ms, with an initial delay of 300 ms, leading to a maximum of 15 spikes ( = action potentials). The postsynaptic conductance, *G*, is described by 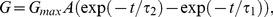
(1)where *G*_max_ is the maximal conductance of the synapse, *A* is set to 1.72 so that the synaptic peak conductance equals *G*_max_ in the present case. *τ*_1_ is the rise time constant ( = 0.5 ms), *τ*_2_ is the decay time constant ( = 3 ms). The reversal potential used was 0 mV. The synaptic conductances were set to increase linearly with the distance from the soma [55], [56] and thus *G*_max_ was set to 2.0, 2.3, 2.5, 3.0, and 3.4 nS respectively.

### Model of synchronicity of the input

To obtain different degrees of synchronicity we used a temporal normal distribution for either 1) a deterministic input model, or 2) a stochastic input model. In both models, the synchronicity level is defined as the standard deviation of the distribution. In the deterministic model, the standard deviation of the distribution determines the time intervals between inputs (see Figure 1D). The time points for five inputs were computed to approximate the probability density function with mean 0 and standard deviation 1. The resulting time points (−0.9674, −0.4307, 0, 0.4307, 0.9674) were in different simulations of a specific synchronicity level multiplied by the synchronicity level value (the standard deviation). When using the stochastic model, five synaptic time points were generated using a pseudo random number generator with the probability density function of mean 0 and standard deviation given by the synchronicity level. As the output for a given synchronicity level in response to an input would be a spike or no spike, output spike probability was calculated by counting the number of spikes produced over the 50 repetitions and normalizing by the maximum possible (50). The stochastic simulation was repeated 50 times to generate stable mean values of output spike probability. However, the stochastic simulations were relatively time demanding, and because the results were similar for the two models, we used the deterministic model in this study except when otherwise noted. It is important to note that for the region of largest relevance for this study, the highest level of synchronicity, the two models produced similar results.

### Pathological models of epilepsy

To generate pathological models, we either increased the Na conductance by 22% [31] (the “Increased Na current pathology model”), or decreased the K_A_ conductance by 50% [3] (the “Decreased K_A_ current pathology model”).

### PUFA modulation of voltage-gated ion channels

As mentioned in the Introduction, PUFAs modulate voltage-gated ion channels by shifting the steady-state activation and/or inactivation curves in negative direction along the voltage axis (Fig. 1B). To convert shifts, ΔV, to concentrations, c, and vice versa we used the general dose-response curve (as used in e.g. [24], [35]):
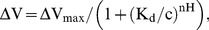
(2)where ΔV_max_ is the maximal shift, K_d_ is the dissociation constant, and n_H_ is the Hill coefficient. In Na channels, PUFAs mainly affect the steady-state inactivation curve, leading to a reduced Na current, and in K channels PUFAs mainly affect the steady-state activation curve, leading to an increased K current. To implement the effects on the Na channel we used the data from rat CA1 Na channels [35], where ΔV_max_  = −11.2 mV, K_d_  = 2.1 μM, and n_H_  = 2.0. To implement the effects on the K_A_ channel we used the data from the Shaker K channel expressed in *Xenopus* oocytes [24], where ΔV_max_  = −9.6 mV, K_d_  = 79 μM, and n_H_  = 1.

The *Xenopus* oocyte expression system is known to underestimate the efficacy of pharmacological substances [48]. Therefore, in the quantitative evaluation we will also give alternative therapeutic intervals assuming a 10 times higher affinity in CA1 neurons than in the *Xenopus* oocytes (referred to as CA1 affinity). In some of the simulations we shifted steady-state inactivation and activation curves equally much. The dynamics of K_A_ is different in proximal dendrites compared with distal dendrites [57]. When implementing the PUFA shift the distal and proximal K_A_ steady-state gates were shifted equally. PUFAs (docosahexaenoic acid, eicosapentaenoic acid, or arachidonic acid) hyperpolarize the resting membrane potential of excitable cells [37]–[39]. We implemented the modulation of the resting membrane potential as a hyperpolarizing shift of the resting potential of up to −4 mV by increasing the conductance of a leak channel. A concentration of about 10 μM PUFA shifts the resting potential in negative direction with about −4 mV (shift range from −1.5 to −5 mV), but no dose-response curve is given. However, assuming a Hill coefficient of 1 (Eq. 2), the maximum concentration (c_max_) required for a shift in resting potential (ΔV_M_) is c_max_  = −10 ΔV_M_/4. For example, the maximum concentration to alter the resting potential with −1 mV is 2.5 μM PUFA.

### Functional correction of the pathological models

We defined the pathological model as functionally corrected if it generated zero spikes for completely synchronized input (0 ms) and the maximum number of spikes (15) at synchronicity level 2 ms. Using this definition, the focus is set on the width of the region of effective suppression at high synchronicity levels, leading to a robust measure avoiding a dependency on the details of this process. The interval of modulatory ion channel change where these two conditions were satisfied is referred to as the therapeutic interval. The effect was expressed in terms of shift of the channel (mV) rather than in concentration of PUFA for reasons discussed in the preceding paragraph. The interval was estimated by binary search which terminated when the interval was less than 0.1 mV.

## References

[1] Meisler MH, Kearney JA (2005) Sodium channel mutations in epilepsy and other neurological disorders. J Clin Invest 115: 2010–2017. 16075041 10.1172/JCI25466 PMC1180547

[2] Hahn A, Neubauer BA (2009) Sodium and potassium channel dysfunctions in rare and common idiopathic epilepsy syndromes. Brain Dev 31: 515–520. 19464834 10.1016/j.braindev.2009.04.012

[3] Singh B, Ogiwara I, Kaneda M, Tokonami N, Mazaki E, et al (2006) A Kv4.2 truncation mutation in a patient with temporal lobe epilepsy. Neurobiol Dis 24: 245–253. 16934482 10.1016/j.nbd.2006.07.001

[4] Biervert C, Schroeder BC, Kubisch C, Berkovic SF, Propping P, et al (1998) A potassium channel mutation in neonatal human epilepsy. Science 279: 403–406. 9430594 10.1126/science.279.5349.403

[5] Singh NA, Charlier C, Stauffer D, DuPont BR, Leach RJ, et al (1998) A novel potassium channel gene, KCNQ2, is mutated in an inherited epilepsy of newborns. Nat Genet 18: 25–29. 9425895 10.1038/ng0198-25

[6] Charlier C, Singh NA, Ryan SG, Lewis TB, Reus BE, et al (1998) A pore mutation in a novel KQT-like potassium channel gene in an idiopathic epilepsy family. Nat Genet 18: 53–55. 9425900 10.1038/ng0198-53

[7] Browne DL, Gancher ST, Nutt JG, Brunt ER, Smith EA, et al (1994) Episodic ataxia/myokymia syndrome is associated with point mutations in the human potassium channel gene, KCNA1. Nat Genet 8: 136–140. 7842011 10.1038/ng1094-136

[8] Hille B (1977) Local anaesthetics: Hydrophilic and hydrophobic pathways for the drug-receptor reaction. JGen Physiol 69: 497–575. 300786 10.1085/jgp.69.4.497 PMC2215053

[9] Ragsdale DS, McPhee JC, Scheuer T, Catterall WA (1996) Common molecular determinants of local anesthetic, antiarrhythmic, and anticonvulsant block of voltage-gated Na+ channels. Proc Natl Acad Sci U S A 93: 9270–9275. 8799190 10.1073/pnas.93.17.9270 PMC38631

[10] Rogawski MA, Loscher W (2004) The neurobiology of antiepileptic drugs. Nat Rev Neurosci 5: 553–564. 15208697 10.1038/nrn1430

[11] Schuele SU, Luders HO (2008) Intractable epilepsy: management and therapeutic alternatives. Lancet Neurol 7: 514–524. 18485315 10.1016/S1474-4422(08)70108-X

[12] Sankar R, Holmes GL (2004) Mechanisms of action for the commonly used antiepileptic drugs: relevance to antiepileptic drug-associated neurobehavioral adverse effects. J Child Neurol 19 Suppl 1S6–14. 15526966 10.1177/088307380401900102

[13] Loring DW, Marino S, Meador KJ (2007) Neuropsychological and behavioral effects of antiepilepsy drugs. Neuropsychol Rev 17: 413–425. 17943448 10.1007/s11065-007-9043-9

[14] Bragin A, Azizyan A, Almajano J, Wilson CL, Engel J Jr (2005) Analysis of chronic seizure onsets after intrahippocampal kainic acid injection in freely moving rats. Epilepsia 46: 1592–1598. 16190929 10.1111/j.1528-1167.2005.00268.x

[15] Bragin A, Mody I, Wilson CL, Engel J Jr (2002) Local generation of fast ripples in epileptic brain. J Neurosci 22: 2012–2021. 11880532 10.1523/JNEUROSCI.22-05-02012.2002 PMC6758883

[16] Bragin A, Wilson CL, Engel J Jr (2000) Chronic epileptogenesis requires development of a network of pathologically interconnected neuron clusters: a hypothesis. Epilepsia 41 Suppl 6S144–152. 10999536 10.1111/j.1528-1157.2000.tb01573.x

[17] Lasztoczi B, Antal K, Nyikos L, Emri Z, Kardos J (2004) High-frequency synaptic input contributes to seizure initiation in the low-[Mg2+] model of epilepsy. Eur J Neurosci 19: 1361–1372. 15016094 10.1111/j.1460-9568.2004.03231.x

[18] Gasparini S, Migliore M, Magee JC (2004) On the initiation and propagation of dendritic spikes in CA1 pyramidal neurons. J Neurosci 24: 11046–11056. 15590921 10.1523/JNEUROSCI.2520-04.2004 PMC6730267

[19] Azouz R, Gray CM (1999) Cellular mechanisms contributing to response variability of cortical neurons in vivo. J Neurosci 19: 2209–2223. 10066274 10.1523/JNEUROSCI.19-06-02209.1999 PMC6782570

[20] Losonczy A, Makara JK, Magee JC (2008) Compartmentalized dendritic plasticity and input feature storage in neurons. Nature 452: 436–441. 18368112 10.1038/nature06725

[21] Fransén E, Tigerholm J (2010) Role of A-type potassium currents in excitability, network synchronicity, and epilepsy. Hippocampus 20: 877–887. 19777555 10.1002/hipo.20694 PMC3222850

[22] Fraser DD, Whiting S, Andrew RD, Macdonald EA, Musa-Veloso K, et al (2003) Elevated polyunsaturated fatty acids in blood serum obtained from children on the ketogenic diet. Neurology 60: 1026–1029. 12654976 10.1212/01.wnl.0000049974.74242.c6

[23] Leaf A, Xiao YF, Kang JX, Billman GE (2005) Membrane effects of the n-3 fish oil fatty acids, which prevent fatal ventricular arrhythmias. J Membr Biol 206: 129–139. 16456723 10.1007/s00232-005-0789-9

[24] Xu XP, Erichsen D, Börjesson SI, Dahlin M, Åmark P, et al (2008) Polyunsaturated fatty acids and cerebrospinal fluid from children on the ketogenic diet open a voltage-gated K channel: A putative mechanism of antiseizure action. Epilepsy Res 80: 57–66. 18448313 10.1016/j.eplepsyres.2008.03.013

[25] Taha AY, Burnham WM, Auvin S (2010) Polyunsaturated fatty acids and epilepsy. Epilepsia 51: 1348–1358. 20608961 10.1111/j.1528-1167.2010.02654.x

[26] Boland LM, Drzewiecki MM (2008) Polyunsaturated fatty acid modulation of voltage-gated ion channels. Cell Biochem Biophys 52: 59–84. 18830821 10.1007/s12013-008-9027-2

[27] Börjesson SI, Hammarström S, Elinder F (2008) Lipoelectric modification of ion channel voltage gating by polyunsaturated fatty acids. Biophys J 95: 2242–2253. 18502799 10.1529/biophysj.108.130757 PMC2517024

[28] Börjesson SI, Parkkari T, Hammarström S, Elinder F (2010) Electrostatic Tuning of Cellular Excitability. Biophys J 98: 396–403. 20141752 10.1016/j.bpj.2009.10.026 PMC2814211

[29] Börjesson SI, Elinder F (2011) An electrostatic potassium channel opener targeting the final voltage sensor transition. J Gen Physiol 137: 563–577. 21624947 10.1085/jgp.201110599 PMC3105513

[30] Buzsaki G (1986) Hippocampal sharp waves: their origin and significance. Brain Res 398: 242–252. 3026567 10.1016/0006-8993(86)91483-6

[31] Vreugdenhil M, Faas GC, Wadman WJ (1998) Sodium currents in isolated rat CA1 neurons after kindling epileptogenesis. Neuroscience 86: 99–107. 9692746 10.1016/s0306-4522(98)00041-4

[32] Castro PA, Cooper EC, Lowenstein DH, Baraban SC (2001) Hippocampal heterotopia lack functional Kv4.2 potassium channels in the methylazoxymethanol model of cortical malformations and epilepsy. J Neurosci 21: 6626–6634. 11517252 10.1523/JNEUROSCI.21-17-06626.2001 PMC6763091

[33] Xiao YF, Kang JX, Morgan JP, Leaf A (1995) Blocking effects of polyunsaturated fatty acids on Na+ channels of neonatal rat ventricular myocytes. Proc Natl Acad Sci U S A 92: 11000–11004. 7479925 10.1073/pnas.92.24.11000 PMC40558

[34] Xiao YF, Wright SN, Wang GK, Morgan JP, Leaf A (1998) Fatty acids suppress voltage-gated Na+ currents in HEK293t cells transfected with the alpha-subunit of the human cardiac Na+ channel. Proc Natl Acad Sci U S A 95: 2680–2685. 9482947 10.1073/pnas.95.5.2680 PMC19460

[35] Vreugdenhil M, Bruehl C, Voskuyl RA, Kang JX, Leaf A, et al (1996) Polyunsaturated fatty acids modulate sodium and calcium currents in CA1 neurons. Proc Natl Acad Sci U S A 93: 12559–12563. 8901621 10.1073/pnas.93.22.12559 PMC38031

[36] Leaf A, Xiao YF, Kang JX, Billman GE (2003) Prevention of sudden cardiac death by n-3 polyunsaturated fatty acids. Pharmacol Ther 98: 355–377. 12782244 10.1016/s0163-7258(03)00039-1

[37] Lauritzen I, Blondeau N, Heurteaux C, Widmann C, Romey G, et al (2000) Polyunsaturated fatty acids are potent neuroprotectors. Embo J 19: 1784–1793. 10775263 10.1093/emboj/19.8.1784 PMC302016

[38] Xiao Y, Li X (1999) Polyunsaturated fatty acids modify mouse hippocampal neuronal excitability during excitotoxic or convulsant stimulation. Brain Res 846: 112–121. 10536218 10.1016/s0006-8993(99)01997-6

[39] Kang JX, Xiao YF, Leaf A (1995) Free, long-chain, polyunsaturated fatty acids reduce membrane electrical excitability in neonatal rat cardiac myocytes. Proc Natl Acad Sci U S A 92: 3997–4001. 7732020 10.1073/pnas.92.9.3997 PMC42089

[40] Bough KJ, Rho JM (2007) Anticonvulsant mechanisms of the ketogenic diet. Epilepsia 48: 43–58. 17241207 10.1111/j.1528-1167.2007.00915.x

[41] Tanner GR, Lutas A, Martinez-Francois JR, Yellen G (2011) Single K ATP channel opening in response to action potential firing in mouse dentate granule neurons. J Neurosci 31: 8689–8696. 21653873 10.1523/JNEUROSCI.5951-10.2011 PMC3133530

[42] Migliore M, Novara G, Tegolo D (2008) Single neuron binding properties and the magical number 7. Hippocampus 18: 1122–1130. 18680161 10.1002/hipo.20480

[43] Bernard C, Anderson A, Becker A, Poolos NP, Beck H, et al (2004) Acquired dendritic channelopathy in temporal lobe epilepsy. Science 305: 532–535. 15273397 10.1126/science.1097065

[44] Francis J, Jugloff DG, Mingo NS, Wallace MC, Jones OT, et al (1997) Kainic acid-induced generalized seizures alter the regional hippocampal expression of the rat Kv4.2 potassium channel gene. Neurosci Lett 232: 91–94. 9302094 10.1016/s0304-3940(97)00593-4

[45] Tigerholm J, Fransén E (2011) Reversing nerve cell pathology by optimizing modulatory action on target ion channels. Biophys J 101: 1871–1879. 22004740 10.1016/j.bpj.2011.08.055 PMC3192983

[46] Hong MP, Kim HI, Shin YK, Lee CS, Park M, et al (2004) Effects of free fatty acids on sodium currents in rat dorsal root ganglion neurons. Brain Res 1008: 81–91. 15081385 10.1016/j.brainres.2004.02.033

[47] Zagotta WN, Aldrich RW (1990) Voltage-dependent gating of Shaker A-type potassium channels in Drosophila muscle. J Gen Physiol 95: 29–60. 2299331 10.1085/jgp.95.1.29 PMC2216290

[48] Rolf S, Haverkamp W, Borggrefe M, Musshoff U, Eckardt L, et al (2000) Effects of antiarrhythmic drugs on cloned cardiac voltage-gated potassium channels expressed in Xenopus oocytes. Naunyn Schmiedebergs Arch Pharmacol 362: 22–31. 10935529 10.1007/s002100000257

[49] Azouz R, Gray CM (2003) Adaptive coincidence detection and dynamic gain control in visual cortical neurons in vivo. Neuron 37: 513–523. 12575957 10.1016/s0896-6273(02)01186-8

[50] Xiong Q, Sun H, Li M (2007) Zinc pyrithione-mediated activation of voltage-gated KCNQ potassium channels rescues epileptogenic mutants. Nat Chem Biol 3: 287–296. 17435769 10.1038/nchembio874

[51] Wuttke TV, Seebohm G, Bail S, Maljevic S, Lerche H (2005) The new anticonvulsant retigabine favors voltage-dependent opening of the Kv7.2 (KCNQ2) channel by binding to its activation gate. Mol Pharmacol 67: 1009–1017. 15662042 10.1124/mol.104.010793

[52] Blom SM, Schmitt N, Jensen HS (2009) The acrylamide (S)-2 as a positive and negative modulator of Kv7 channels expressed in Xenopus laevis oocytes. PLoS One 4: e8251. 20011514 10.1371/journal.pone.0008251 PMC2788219

[53] Peretz A, Pell L, Gofman Y, Haitin Y, Shamgar L, et al (2010) Targeting the voltage sensor of Kv7.2 voltage-gated K+ channels with a new gating-modifier. Proc Natl Acad Sci U S A 107: 15637–15642. 20713704 10.1073/pnas.0911294107 PMC2932606

[54] Hines ML, Carnevale NT (1997) The NEURON simulation environment. Neural Comput 9: 1179–1209. 9248061 10.1162/neco.1997.9.6.1179

[55] Pettit DL, Augustine GJ (2000) Distribution of functional glutamate and GABA receptors on hippocampal pyramidal cells and interneurons. J Neurophysiol 84: 28–38. 10899180 10.1152/jn.2000.84.1.28

[56] Magee JC, Cook EP (2000) Somatic EPSP amplitude is independent of synapse location in hippocampal pyramidal neurons. Nat Neurosci 3: 895–903. 10966620 10.1038/78800

[57] Hoffman DA, Magee JC, Colbert CM, Johnston D (1997) K+ channel regulation of signal propagation in dendrites of hippocampal pyramidal neurons. Nature 387: 869–875. 9202119 10.1038/43119

[58] Segev I, London M (2000) Untangling Dendrites with Quantitative Models. Science 290: 744–750. 11052930 10.1126/science.290.5492.744

[59] Gasparini S, Migliore M, Magee J (2004) On the Initiation and Propagation of Dendritic Spikes in CA1 Pyramidal Neurons. J. Neurosci. 24: 11046–11056. 10.1523/JNEUROSCI.2520-04.2004 PMC6730267 15590921

[60] Branco T, Clark BA, Häusser M (2010) Dendritic discrimination of temporal input sequences in cortical neurons. Science. 329: 1671–1675. 10.1126/science.1189664 PMC6354899 20705816

[61] Pfeuty B, Mato G, Golomb D, Hansel D (2003) Electrical synapses and synchrony: the role of intrinsic currents. J Neurosci. 23: 6280–94. 10.1523/JNEUROSCI.23-15-06280.2003 PMC6740557 12867513

[62] Pfeuty B, Mato G, Golomb D, Hansel D (2005) The combined effects of inhibitory and electrical synapses in synchrony. Neural Comput. 17: 633–70. 10.1162/0899766053019917 15802009

[63] Cymbaluk GS, Nikolaev EV, Borisyuk RM (1994) In-phase and antiphase oscillations in a model of two electrically-coupled pacemakers. Biol. Cyb. 71: 153–160. 10.1007/BF00197318 8068776

[64] Sherman A, Rinzel J (1992) Rhythmogenic effects of weak electrotonic coupling in neuronal models. Proc Natl Acad Sci U S A. 89: 2471–4. 10.1073/pnas.89.6.2471 PMC48680 1549611

[65] Takekawa T, Aoyagi T, Fukai T (2007) Synchronous and asynchronous bursting states: role of intrinsic neural dynamics. J Comput Neurosci. 23: 189–200. 10.1007/s10827-007-0027-9 17387606

[66] Smeal RM, Ermentrout GB, White JA (2010) Phase-response curves and synchronized neural networks. Philos Trans R Soc Lond B Biol Sci. 365: 2407–22. 10.1098/rstb.2009.0292 PMC2894948 20603361

